# Knowledge domain and hotspots concerning photosensitive hydrogels for tissue engineering applications: A bibliometric and visualized analysis (1996-2022)

**DOI:** 10.3389/fbioe.2022.1067111

**Published:** 2022-11-14

**Authors:** Hongxun Fu, Baojun Yu, Hao Wang, Haibin Tong, Lin Jiang, Yupeng Zhang, Guixian Meng, Meiyan Sun, Jieqiong Lin

**Affiliations:** ^1^ Key Laboratory of Micro/Nano and Ultra-precision Manufacturing, Jilin Province, School of Mechatronic Engineering, Changchun University of Technology, Changchun, China; ^2^ College of Laboratory Medicine, Jilin Medical University, Jilin, China; ^3^ College of Life and Environmental Science, Wenzhou University, Wenzhou, China; ^4^ Affiliated Hospital of Beihua University, Jilin, China

**Keywords:** photosensitive hydrogels, tissue engineering, bibliometrics, bibliometrix, citespace

## Abstract

**Objective:** The aim of tissue engineering (TE) is to replace the damaged tissues or failed organs, or restore their missing functions. The important means to achieve this aim is to integrate biomaterials and life elements. Hydrogels are very attractive biomaterials in the field of TE. In particular, engineering extracellular matrices (ECMs) formed by photosensitive hydrogels have captivated much attention, because photopolymerization has many advantages over traditional polymerization approaches, such as rapidity of reaction, spatiotemporal controllability of polymerization process, and operability at physiological temperature, especially it can realize the fabrications of engineering ECMs in the presence of living cells. There have been many excellent reviews on the applications of photosensitive hydrogels in TE in recent years, however, it is inevitable that researchers may have left out many important facts due to exploring the literature from one or a few aspects. It is also a great challenge for researchers to explore the internal relationships among countries, institutions, authors, and references from a large number of literatures in related fields. Therefore, bibliometrics may be a powerful tool to solve the above problems. A bibliometric and visualized analysis of publications concerning the photosensitive hydrogels for TE applications was performed, and the knowledge domain, research hotspots and frontiers in this topic were identified according to the analysis results.

**Methods:** We identified and retrieved the publications regarding the photosensitive hydrogels for TE applications between 1996 and 2022 from Web of Science Core Collection (WoSCC). Bibliometric and visualized analysis employing CiteSpace software and R-language package Bibliometrix were performed in this study.

**Results:** 778 publications meeting the eligibility criteria were identified and retrieved from WoSCC. Among those, 2844 authors worldwide participated in the studies in this field, accompanied by an average annual article growth rate of 15.35%. The articles were co-authored by 800 institutions from 46 countries/regions, and the United States published the most, followed by China and South Korea. As the two countries that published the most papers, the United States and China could further strengthen cooperation in this field. Univ Colorado published the most articles (*n* = 150), accounting for 19.28% of the total. The articles were distributed in 112 journals, among which Biomaterials (*n* = 66) published the most articles, followed by Acta Biomaterialia (*n* = 54) and Journal of Biomedical Materials Research Part A (*n* = 42). The top 10 journals published 47.8% of the 778 articles. The most prolific author was Anseth K (*n* = 33), followed by Khademhosseini A (*n* = 29) and Bryant S (*n* = 22). A total of 1443 keywords were extracted from the 778 articles and the keyword with the highest centrality was “extracellular matrix” (centrality: 0.12). The keywords appeared recently with strong citation bursts were “gelatin”, “3d printing” and “3d bioprinting”, representing the current research hotspots in this field. “Gelma”, “3d printing” and “thiol-ene” were the research frontiers in recent years.

**Conclusion:** This bibliometric and visualized study offered a comprehensive understanding of publications regarding the photosensitive hydrogels for TE applications from 1996 to 2022, including the knowledge domain, research hotspots and frontiers in this filed. The outcome of this study would provide insights for scholars in the related research filed.

## Introduction

The aim of tissue engineering (TE) is to replace the damaged tissues or failed organs, or restore their missing functions (Mooney and Langer 2000; Ratner and Bryant, 2004). The important means to achieve this aim is to integrate biomaterials and life elements (seed cells and growth factor) (Mikos et al., 2006; Qin et al., 2014). The tissue substitutes, wound dressings, or substrates for TE all put forward various requirements for biomaterials. In particular, the engineering extracellular matrices (ECMs), or the mimics of the microenvironment for cell growth, and 3D cell culture models, are usually called the tissue engineering scaffolds, which interact with cells as ECMs before forming new tissues (Yao et al., 2013; Jafari et al., 2017; Jing et al., 2022). TE scaffold is the crux to achieve the goal of TE (Langer and Vacanti 1993; Wu et al., 2020). In order to provide engineering ECMs for cells to produce new tissues, scaffolds have to possess biocompatibility, proper degradability, reasonable mechanical properties, and sufficient porosity and pore connectivity (Zhang et al., 2019). In addition, integrating different functions such as hydrophilicity, biological and physical cues could promote the formation of new tissues (Jing et al., 2022). Hydrogels are three-dimensional (3D) natural or synthetic crosslinked networks composed of polymerization chains formed by hydrophilic monomers or macromers (Qin et al., 2014; Gholamali and Yadollahi 2021). Due to the ability to simulate many properties of natural ECMs, hydrogels have been widely used in biomedical fields, such as drug delivery and tissue engineering (Fu et al., 2022; Jing et al., 2022). Especially for TE, hydrogel scaffolds have captivated much attention because they provide cells with not only the mechanical supports but also the interconnected pores for permeating nutrients, oxygen and metabolite (Qin et al., 2014). In addition, the physical properties of hydrogels can be adjusted and they can be functionalized to meet the requirements of different tissues (Qin et al., 2014; Zhao et al., 2020). Hydrogels can be obtained by many polymerization strategies such as heat and redox, however, photochemistry is the most interesting to researchers in this field (Qin et al., 2014; Zhang et al., 2019). Photoinitiator(PI) molecules in the photosensitive hydrogel precursors absorb photons to produce free radicals, which then initiate radical polymerization and constitute crosslinked hydrogel networks. Photon absorption of PIs can be either single photon or multi photons (Jing et al., 2022). Photopolymerization has many advantages over traditional polymerization approaches, such as rapidity of reaction, spatiotemporal controllability of polymerization process, operability at physiological temperature, etc., especially it can realize scaffold fabrications in the presence of living cells (Qin et al., 2014).

Since the photosensitive hydrogels applied in TE were first described in 1996, they have shown a very promising potential in this field. There have been many excellent reviews on the applications of photosensitive hydrogels in TE in recent years, however, it is inevitable that researchers may have left out many important facts due to exploring the literature from one or a few aspects. It is also a great challenge for researchers to explore the internal relationships among countries, institutions, authors, and references from a large number of literatures in related fields. Therefore, bibliometrics may be a powerful tool to solve the above problems.

As a statistical method combining quantitative and qualitative analysis, bibliometrics explores literature from multiple aspects, such as countries, institutions, journals, authors, keywords and cited literature, to identify the knowledge base, hotspots, frontiers, development trends of related research fields, and relationships between specific research areas. (Chen 2004; Chen 2017; Zhang et al., 2021; Fu et al., 2022). With the continuous development of this discipline, bibliometrics has creatively visualized the information regarding the publications to form network maps, which assist researchers in intuitively grasping the important connotation and denotation implied in the literature (Ellegaard and Wallin 2015; Gonzalez-Alcaide et al., 2017). Bibliometrics has extended to analyze a multitude of scientific research fields, and has obtained a lot of achievements (Ugolini et al., 2010; Wei et al., 2016; Ke et al., 2020; Gu et al., 2021; Shi et al., 2021). However, the bibliometric study for photosensitive hydrogels is still lacking, especially their applications in TE.

In this article, we conducted bibliometric and visualized study using bibliometrics software CiteSpace and the R-language package Bibliometrix to analyze the retrieved publications worldwide regarding the applications of photosensitive hydrogels for TE, and the knowledge domain, research hotspots and frontiers in this topic were identified according to the analysis results.

## Materials and methods

### Data source and search strategy

We performed a targeted search of literature in Web of Science Core Collection (WoSCC) in the field of photosensitive hydrogels applications for TE from 1 January 1996 (the first year photosensitive hydrogels for TE were reported) to 1 October 2022 (end date of the search). WoSCC is a very commonly used database for bibliometric analysis. Following concepts were used to conduct the queries: (((TS= (photopolymeriz* hydrogel*)) OR TS= (photocrosslink* hydrogel*)) OR TS= (photosensitive hydrogel*)) AND TS= (“tissue engineer*") and the English literature types were filtered as “article”. The qualified literature was selected for further bibliometric analysis, and the exported record content was “full record and cited references”. After removing duplicates, topics were screened for any mention of photosensitive hydrogels for TE applications by independent reviewers. All literature search and information extraction work were completed continuously within one day to avoid data deviation due to date change.

### Statistical analysis

CiteSpace (version 6.1. R3), and the R-language package Bibliometrix 4.2.1 were used to conduct the bibliometric and visualized analysis in this study. Information was extracted and analyzed by automatic algorithm and machine intelligence of bibliometrics software.

Bibliometrix is an R-language tool for comprehensive science mapping analysis. It has superior flexibility and can be facilely upgraded and operated (Aria and Cuccurullo, 2017). Using Bibliometrix, we obtained the data of overall information, trend topics, cited references, landmark literature regarding the publications of the research field, and conducted countries analysis, journals analysis, institutions analysis and authors productivity analysis.

CiteSpace is an analysis software developed for bibliometrics, which can be utilized to analyze the knowledge base, research content, hotpots and frontiers of specific research fields by visualizing network maps (Chen 2004; Chen and Leydesdorff 2014). In this article, CiteSpace was used to obtain dual-map overlay of citations and perform the analysis for keywords, references, clusters, and the collaboration relationships among countries and institutions. It is noteworthy to mention that modularity Q and mean silhouette value are two vital evaluation indicators regarding clustering analysis. When the values of these two indicators are greater than 0.3 and 0.5 respectively, the clustering results are considered to be reliable and significant.

## Results

### Overall information of publications

**FIGURE 1:**
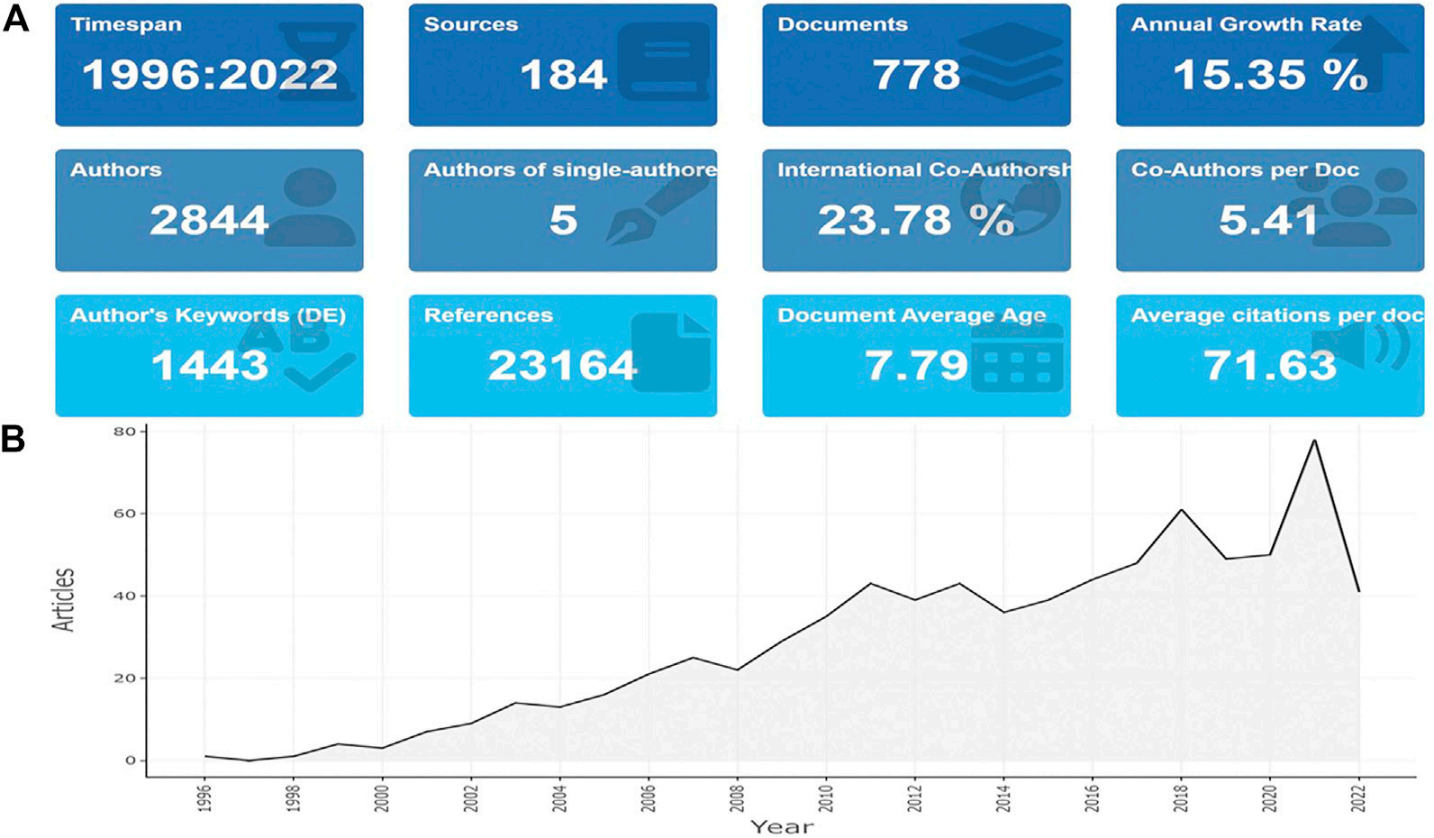
Main information **(A)** and annual scientific production **(B)** of publications concerning the photosensitive hydrogels for TE applications.

We identified and retrieved 778 publications in total met the eligibility criteria from WoSCC. Figure 1A shows the main information of the publications regarding the applications of photosensitive hydrogels for TE. As can be seen from this figure, 2844 authors worldwide participated in the studies in this field we analyzed, accompanied by an average annual article growth rate of 15.35%. Meanwhile, we could confirm that photosensitive hydrogels applications for TE is a relatively new research field due to the 7.79 years of publication average age. As shown in Figure 1B, the number of annual publications generally increased in a time-dependent manner since 1996 which peaked in year 2021. Especially in the past decade, the number of publications in this field showed a rapid growth trend, accounting for 63.75% of the total. The annual scientific production map (Figure 1B) shows that 60 articles were expected to be published in 2022.

### Analysis of countries/regions and institutions

**FIGURE 2:**
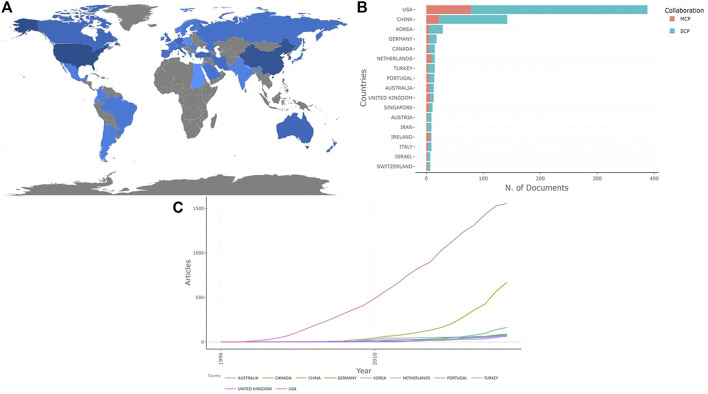
Contributions of different countries related to the research of photosensitive hydrogels for TE applications. **(A)** Global country scientific production contributions (The depth of color represents the number of articles published); **(B)** Top 10 countries with the most artilcles related to photosensitive hydrogels for TE applications (based on the countries where the corresponding authors come from); **(C)** Production of the top 10 countries with the highest productivity over time.

The publications we retrieved were co-authored by 800 institutions from 46 countries/regions. The global distribution of country scientific production regarding the field is shown in Figure 2A. The top 10 countries with the highest number of publications come from four continents, including one country in Oceania (Australia), two in Asia (China and South Korea), two in North America (the USA and Canada) and five in Europe (Germany, Portugal, Turkey, Netherlands and the UK), all of them are developed countries except China and Turkey. Figure 2B and Table 1 show the ranking of the publications number based on the countries where the corresponding authors come from. Among the top 10 countries, the USA published the most articles, followed by China and South Korea. In addition, the United States is not only the first country to engage in the study of photosensitive hydrogels for TE, but also had an absolute advantage in the number of papers increased and published compared with other countries (Figure 2C).In addition, The average citation rate of articles from the top 10 countries with the most published articles shows that the United States not only ranked first in the number of articles, but also had a relatively high citation rate, indicating that its articles are of good quality.

**TABLE 1:** Top 10 countries with the most publications related to photosensitive hydrogels for TE applications (based on the countries where the corresponding authors come from).

Country	Articles	SCP	MCP	Freq	MCP Ratio	Average article citations
USA	388	310	78	0.499	0.201	101.99
CHINA	142	120	22	0.183	0.155	29.92
KOREA	29	24	5	0.037	0.172	33.31
GERMANY	18	13	5	0.023	0.278	54.56
CANADA	15	11	4	0.019	0.267	45.13
NETHERLANDS	15	6	9	0.019	0.6	117.67
TURKEY	15	12	3	0.019	0.2	26.20
PORTUGAL	14	9	5	0.018	0.357	34.57
AUSTRALIA	13	8	5	0.017	0.385	57.00
UNITED KINGDOM	13	6	7	0.017	0.538	38.23
SINGAPORE	11	6	5	0.014	0.455	37.91
AUSTRIA	9	8	1	0.012	0.111	57.67
IRAN	9	7	2	0.012	0.222	24.00
IRELAND	9	3	6	0.012	0.667	25.56
ITALY	9	6	3	0.012	0.333	46.00
ISRAEL	7	5	2	0.009	0.286	71.299
SWITZERLAND	7	5	2	0.009	0.286	123.43

**FIGURE 3:**
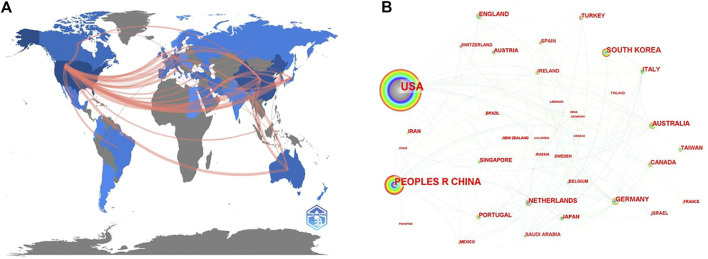
Cooperation of countries regarding the photosensitive hydrogels for TE applications. **(A)** The network map of collaboration relations between countries generated with R-Bibliometrix; **(B)** Visualized network map of collaboration relations between countries generated with CiteSpace.

The visualized international collaboration relation maps were generated by Bibliometrix and CiteSpzce (Figures 3A,B). In these two network maps, there is quite close collaboration between different countries/regions, among which the top 10 countries that published the most articles also have the top 10 largest nodes, indicating that cooperation between countries is conducive to the output of research results. At the same time, we could find that, although the United States and China were the top 2 countries with the most publications, the cooperation between them was not close.

**FIGURE 4:**
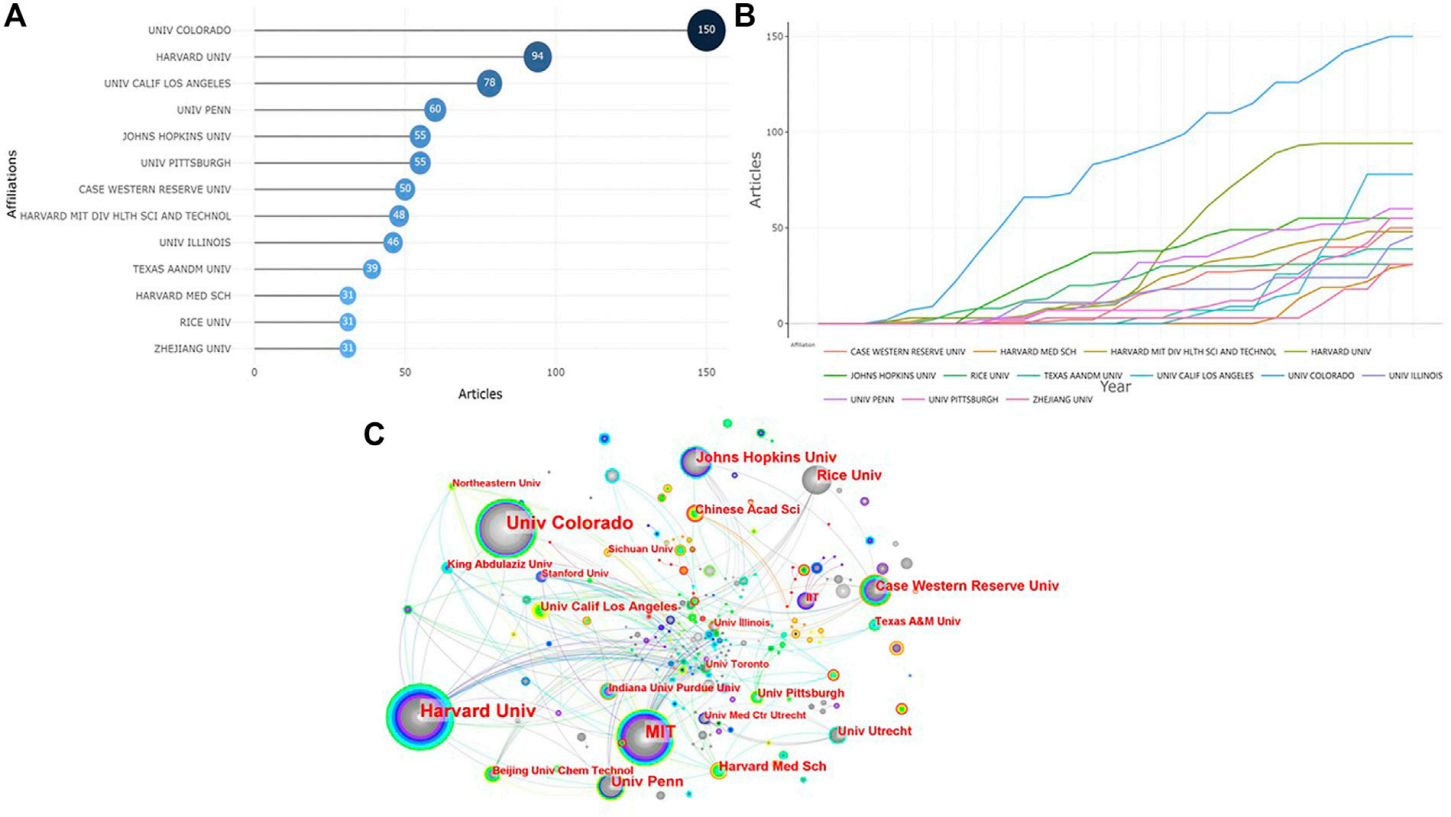
Visualized analysis of institutions concerning the publications of photosensitive hydrogels for TE applications. **(A)** The top 10 institutions with the most published articles; **(B)** Production of the top 10 institutions with the highest productivity over time. **(C)** The network map of collaboration relations between institutions.

Figure 4A demonstrates the top 10 institutions with the highest number of articles regarding the photosensitive hydrogels for TE. Except one of the top 10 institutions comes from China (Zhejiang Univ, ranked 10th), all institutions are located in the United States, of which the Univ Colorado published the most articles (*n* = 150), accounting for 19.28% of the total. Univ Colorado began to study photosensitive hydrogels for TE applications in 1998, and had maintained a high output in the number of publications (Figure 4B). The research in this field did not start in Zhejiang Univ from China until 2006, but the number of articles published by this institution increased relatively rapidly (Figure 4B). As shown in Figure 4C, academic institutions in the United States universally had larger nodes, indicating that they had very close cooperation with other institutions. Several research institutions in China (Chinese Acad Sci, BeiJing Univ Chem Technol and SiChuan Univ) also had relatively large nodes, indicating a relatively significant degree of institutional cooperation. However, there was not much cooperation between them and American research institutions.

### Analysis of authors and journals

**FIGURE 5:**
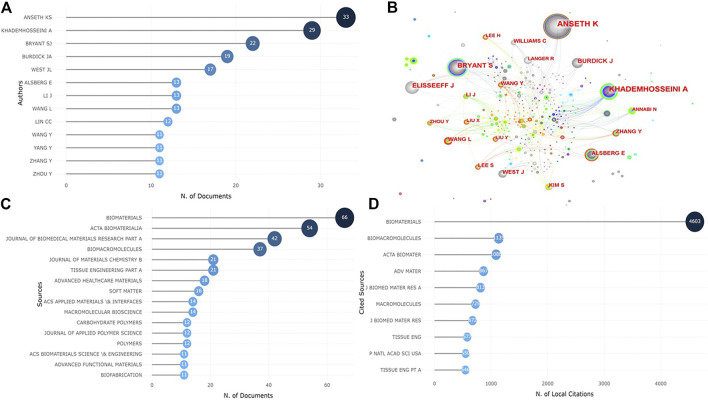
Visualized analysis of authors and journals concerning publications of photosensitive hydrogels for TE applications. **(A)** The top 10 authors with the most published articles; **(B)** The network map of collaboration relations between authors. **(C)** The top 10 most productive journals; **(D)** The top 10 journals with the most local cited publications.

Figure 5A shows that 2844 researchers co-authored the 778 articles, 13 of whom published more than 10 paper. The most prolific author was Anseth K (*n* = 33), followed by Khademhosseini A(*n* = 29) and Bryant S (*n* = 22). In the network map of author collaboration relations (Figure 5B), the large nodes owned by high-yield authors also imply that cooperation contributes to the output of research results.

The publications we analyzed were distributed in 112 journals, among which Biomaterials (*n* = 66) published the most articles, followed by Acta Biomaterialia (*n* = 54) and Journal of Biomedical Materials Research Part A (*n* = 42) (Figure 5C). The top 10 journals published 47.8% of the 778 articles, or 372 articles. In addition, we analyzed the most local cited sources using bibliometrix. Figure 5D shows that the top 3 journals most locally cited were Biomaterials, Biomacromolecules and Acta Biomaterialia.

**FIGURE 6:**
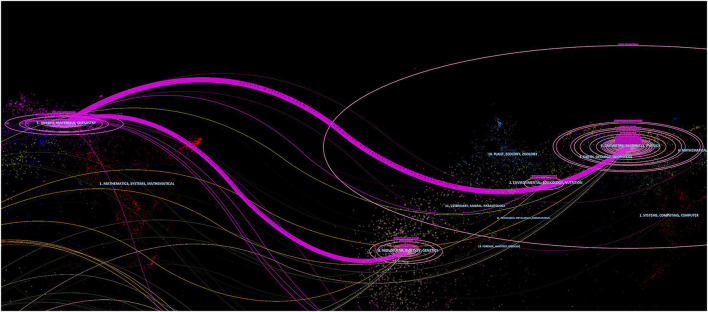
Part of dual-map overlay for journals related to the photosensitive hydrogels for TE applications.

Another unique function of CiteSpace is to generate dual-map, which can show the topic distribution of journals in specific research fields (Chen 2017). In dual-map, the color stripes in the middle connect the two ends of the paths, and the left ends trace the citing journals, the right ends trace the cited journals. There are two main paths in Figure 6, indicating that the publications regarding photosensitive hydrogels for TE applications were mainly involved in Physics/Materials/Chemistry journals, while the cited documents were usually distributed in Chemistry/Materials/Physics and MOLECULAR/BIOLOGY/GENETICS journals.

### Analysis of keywords

**FIGURE 7:**
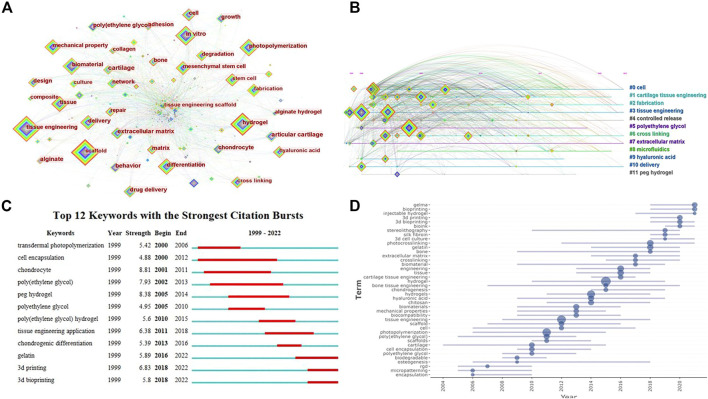
Visualized analysis of keywords regarding the publications on the applications of photosensitive hydrogels for TE. **(A)** The keywords co-occurrence network; **(B)** The timeline of clustering for keywords; **(C)** Keywords burst analysis indicated by the map of “Top 12 Keywords with the Strongest Citation Bursts”; **(D)** Map of keywords trend topics.

A total of 1443 keywords were extracted from the 778 articles. Some top keywords with high frequency and centrality are shown in Table 2. The keyword with the highest centrality was “extracellular matrix” (centrality: 0.12), indicating that it was an important content in this research field. Other significant keywords included “*in vitro*” (frequency:111, centrality: 0.11), “scaffold” (frequency:189, centrality: 0.04), “cartilage” (centrality: 0.11) and “alginate” (centrality: 0.11). The network map of keywords co-occurrence (Figure 7A) shows that “(tissue engineering) scaffold”, “extracellular matrix”, “*in vitro*”, “(mesenchymal) stem cell”, “mechanical property”, “differentiation”, “hyaluronic acid” and “poly(ethylene) glycol” were all remarkable keywords, indicating the main research contents or specific issues in this field. It is worth mentioning here that stem cells, which are special cells that are expected to provide unlimited amounts of cells for transplantation, have been the focus of TE and regenerative medicine in recent years (Akhmanova et al., 2015). Stem cells reside in stem cell niches that are specialized microstructures to control stem cell growth and differentiation by imparting biochemical and biophysical cues (Jing et al., 2022). Using photosensitive hydrogels to fabricate stem cell niches to study cell behaviors *in vitro* is an important content in this field. Alginate, hyaluronic acid and poly(ethylene) glycol are commonly used biomaterials to synthesize photosensitive hydrogels, which are widely used in biomedical fields including TE.

**TABLE 2:** Some typical keywords about the publications related to the photosensitive hydrogels for TE applications.

Rank	keywords	Count	Keywords	Centrality
1	Scaffold	189	Extracellular matrix	0.12
2	Tissue engineering	163	*in vitro*	0.11
3	Hydrogel	154	Cartilage	0.11
4	*in vitro*	111	Alginate	0.11
5	Mechanical property	103	Hydrogel	0.10
6	Tissue	93	Tissue engineering	0.09
7	Biomaterial	93	Tissue	0.09
8	Differentiation	89	Articular cartilage	0.09
9	Photopolymerization	87	Design	0.09
10	Delivery	83	Behavior	0.09

Further, the keywords were clustered and a network map of clustering timeline was formed (Figure 7B). From the network map, we found “PEG hydrogel”, “poly(ethylene) glycol” and “cartilage tissue engineering” were once the research topics in this field, and “cell” and “extracellular matrix” had always been the focuses of research in this field. In addition, although beyond the research content of this article, the applications of photosensitive hydrogels for drug delivery may be another topic, because “delivery” was also a cluster of the keywords. It should be pointed out here that, the mean silhouette value of the cluster was 0.6973 and the modularity Q was 0.3939, showing the clustering is meaningful and reasonable.

Keywords with citation bursts were extracted and Figure 7C shows the top 12 keywords with the strongest citation bursts. “Chondrocyte” was once the most concerned hotspot, due to its strongest citation bursts. In addition, “Poly(ethylene) glycol” and “PEG hydrogel” were all hotspots in this field. The keywords appeared recently with strong citation bursts were “gelatin”, “3d printing” and “3d bioprinting”, representing the current research hotspots in this field. At the same time, we could find from trends of keyword occurrences map (Figure 7D) that “gelma” which is a gelatin derivative with photopolymerizable function, and “bioprinting” were the latest research hotspots in this filed, indicating the potential research direction in the future, which was consistent with the results of keyword bursts analysis.

### Analysis of references

According to the number of citations, the top 10 of 23124 local cited references related to the publications we analyzed are listed in Table 3. An article with 135 citations entitled “Cell-laden microengineered gelatin methacrylate hydrogels” published in the journal of Biomaterials ranked first (29). This paper demonstrated gelatin methacrylate (GelMA) as a hydrogel platform for fabricating cell-responsive microtissues in the presence of living cells, proving GelMA is a kind of cell-responsive microengineered photosensitive hydrogels. The second and third most locally cited references were “Photopolymerizable hydrogels for tissue engineering applications” published in the journal of Biomaterials (Nguyen and West, 2002) and Cytocompatibility of UV and visible light photoinitiating systems on cultured NIH/3T3 fibroblasts *in vitro*” published in Journal of Biomaterials Science (Bryant et al., 2000).

**TABLE 3:** Top 10 local cited references of publications regarding the photosensitive hydrogels for TE applications.

Rank	Cited references	Citations
1	NICHOL JW, 2010, BIOMATERIALS, V31, P5536, DOI 10.1016/J.BIOMATERIALS. 2010.03.064	135
2	NGUYEN KT, 2002, BIOMATERIALS, V23, P4307, DOI 10.1016/S0142-9612(02)00175-8	111
3	BRYANT SJ, 2000, J BIOMAT SCI-POLYM E, V11, P439, DOI 10.1163/156856200743805	90
4	ENGLER AJ, 2006, CELL, V126, P677, DOI 10.1016/J.CELL.2006.06.044	77
5	VAN DEN BULCKE AI, 2000, BIOMACROMOLECULES, V1, P31, DOI 10.1021/BM990017D	75
5	YUE K, 2015, BIOMATERIALS, V73, P254, DOI 10.1016/J.BIOMATERIALS. 2015.08.045	75
6	ELISSEEFF J, 1999, P NATL ACAD SCI USA, V96, P3104, DOI 10.1073/PNAS.96.6.3104	69
7	FAIRBANKS BD, 2009, BIOMATERIALS, V30, P6702, DOI 10.1016/J.BIOMATERIALS. 2009.08.055	68
8	SAWHNEY AS, 1993, MACROMOLECULES, V26, P581, DOI 10.1021/MA00056A005	66
9	DRURY JL, 2003, BIOMATERIALS, V24, P4337, DOI 10.1016/S0142-9612(03)00340-5	65
9	LEE KY, 2001, CHEM REV, V101, P1869, DOI 10.1021/CR000108X	65
10	BURDICK JA, 2002, BIOMATERIALS, V23, P4315, DOI 10.1016/S0142-9612(02)00176-X	64

**FIGURE 8:**
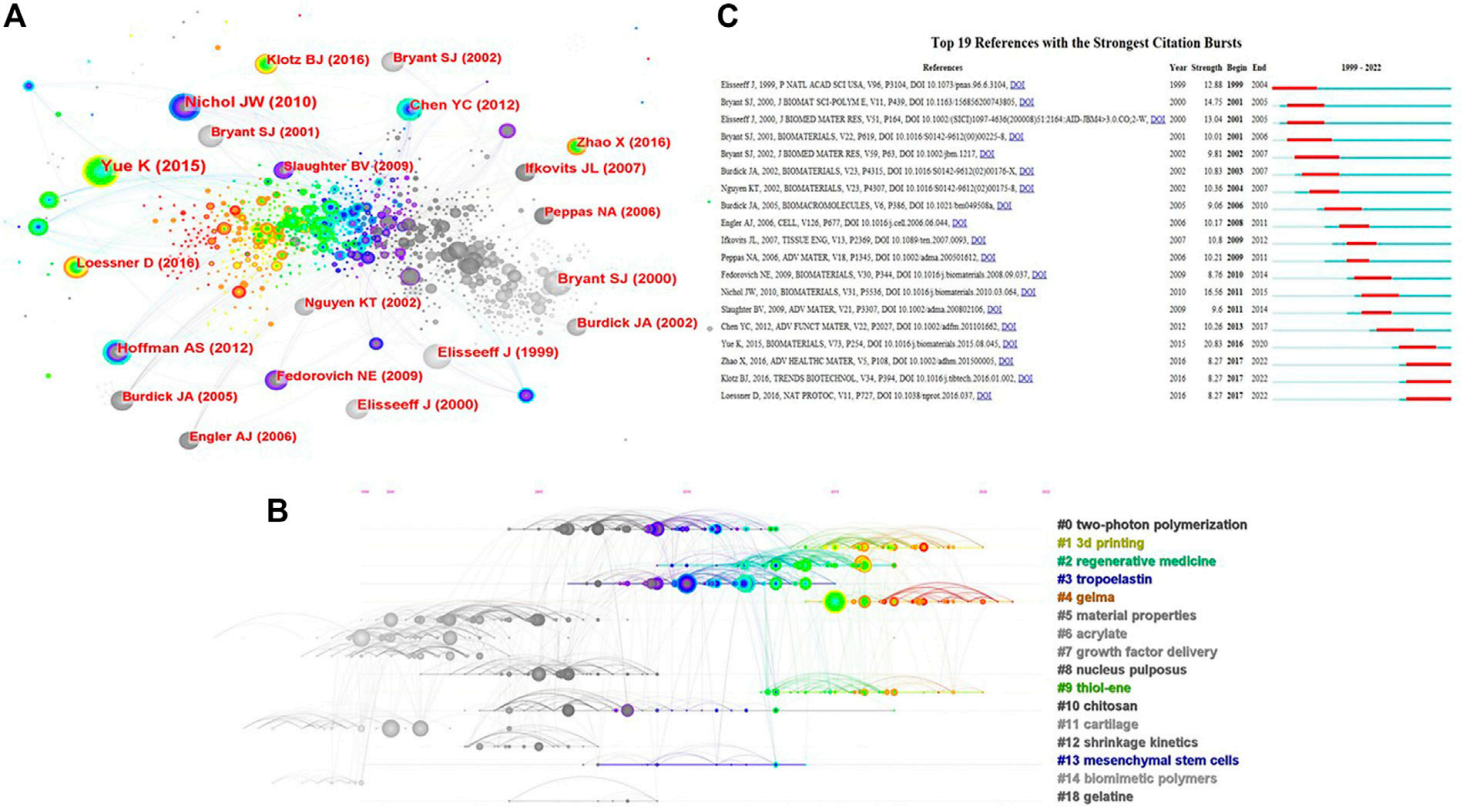
The analysis of references regarding the publications on the applications of photosensitive hydrogels for TE. **(A,B)** The visualized network map and clustering timeline of the co-cited references; **(C)** Top 16 References with the Strongest Citation Bursts.

As presented in Figure 8A, the top 3 co-cited references were (Yue et al., 2015), (Nichol et al., 2010) and (Bryant et al., 2000), of which (Nichol et al., 2010) and (Bryant et al., 2000) were also in the top 3 references with the most local citations. (Yue et al., 2015). is a review focusing on the synthesis and characterization of GelMA and its composites, as well as the fabrication and applications of GelMA-based materials. Moreover, the references were clustered and formed into 19 clusters for clustering analysis. The modularity Q was 0.7962, and the mean silhouette value was 0.8873. The map of clustering timeline (Figure 8B) shows that, “two-photon polymerization”, “regenerative medicine” and “tropoelastin”, etc. were early research areas, while “gelma”, “3d printing” and “thiol-ene” were the research frontiers in recent years. Finally, the references with strong citation bursts were extracted by CiteSpace (Figure 8C). The three publications recently appeared in the top 19 references with the strongest citation bursts were “Photocrosslinkable Gelatin Hydrogel for Epidermal Tissue Engineering” (Zhao et al., 2016), “Gelatin-Methacryloyl Hydrogels: Towards Biofabrication-Based Tissue Repair” (Klotz et al., 2016) and “Functionalization, preparation and use of cell-laden gelatin methacryloyl–based hydrogels as modular tissue culture platforms” (Loessner et al., 2016). These three papers are all reviews discussing the applications of (photopolymerizable) gelatin derivatives for TE, indicating the current research hot spot regarding the photosensitive hydrogels for TE applications.

## Discussion

**FIGURE 9:**
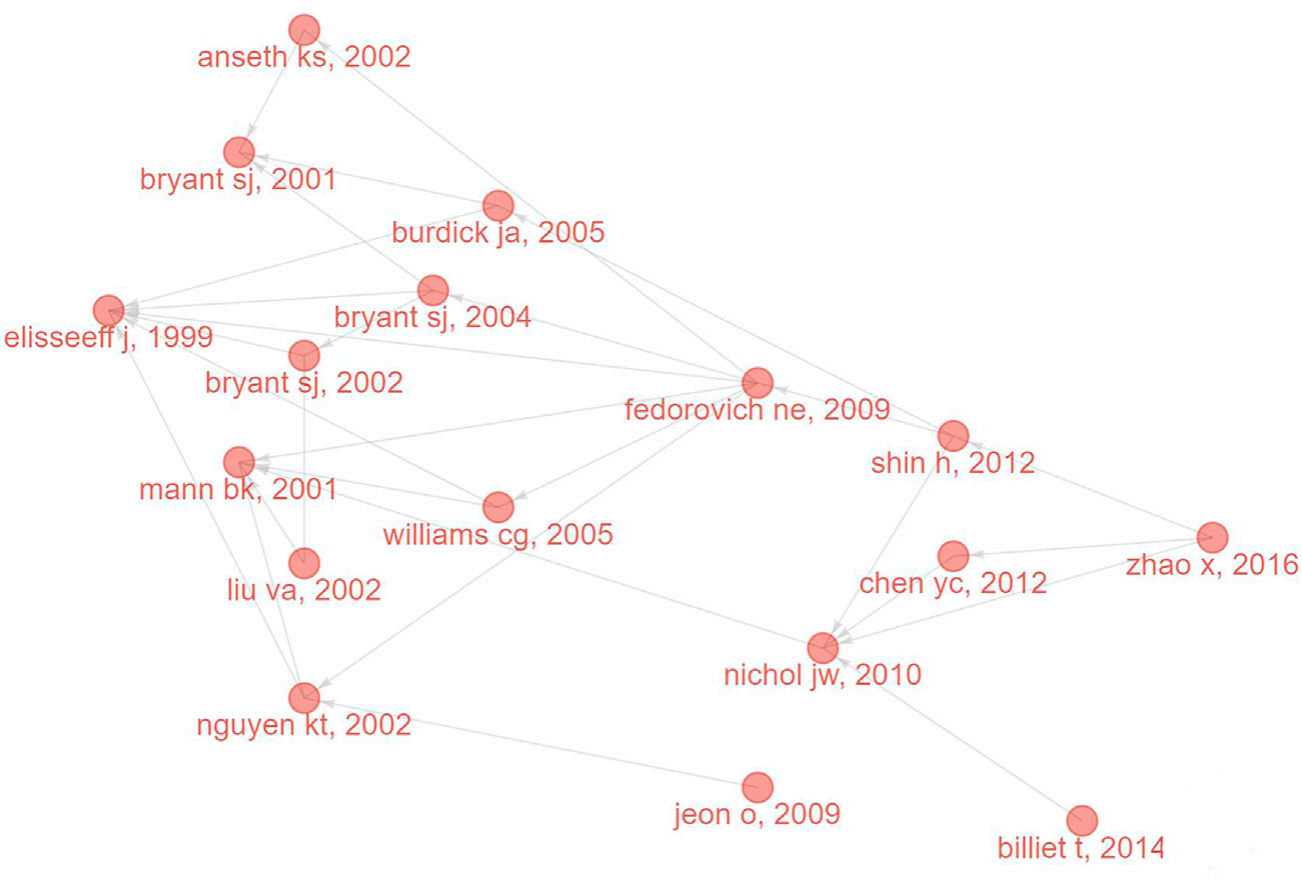
Landmark articles related to the photosensitive hydrogels for TE applications.

In this article, we retrieved and conducted analysis on 778 publications that scoped on the photosensitive hydrogels for TE applications. Some landmark literature was also extracted (Figure 9; Table 4). Since the first paper on the applications of photosensitive hydrogels for TE was published in 1996 (Hubbell et al., 1996), the research field has attracted the attention from scholars. For example, in 1999, a groundbreaking work described the poly (ethylene oxide)-based injectable hydrogels used for TE by transdermal photopolymerization (Elisseeff et al., 1999). Subsequently, Bryant et al. used photocrosslinked poly(ethylene oxide) hydrogels to fabricated tissue engineered cartilage scaffolds encapsulating cells and studied the effects of scaffold thickness on the engineered cartilage (Bryant and Anseth, 2001). With the continuous development of this research field, different types of photosensitive hydrogels have been studied and applied to TE, such as PEG-based hydrogels (Mann et al., 2001), alginate hydrogels (Jeon et al., 2009), gelatin-based hydrogels (Billiet et al., 2014), hyaluronic acid hydrogels (Zerobin et al., 2020) and silk fibroin hydrogels (Hong et al., 2020). The application of photosensitive hydrogels has brought unprecedented promising for the TE and regenerative medicine.

**TABLE 4:** Landmark papers concerning publications related to the photosensitive hydrogels for TE applications.

Paper	Year	LCS	GCS
ELISSEEFF J, 1999, PLAST RECONSTR SURG DOI 10.1097/00006534-199909040-00017	1999	35	165
MANN BK, 2001, BIOMATERIALS DOI 10.1016/S0142-9612(01)00051-5	2001	55	546
BRYANT SJ, 2001, BIOMATERIALS DOI 10.1016/S0142-9612(00)00225-8	2001	25	231
ANSETH KS, 2002, J CONTROL RELEASE DOI 10.1016/S0168-3659(01)00500-4	2002	29	363
NGUYEN KT, 2002, BIOMATERIALS DOI 10.1016/S0142-9612(02)00175-8	2002	111	1205
LIU VA, 2002, BIOMED MICRODEVICES DOI 10.1023/A:1020932105236	2002	26	322
BRYANT SJ, 2002, J BIOMED MATER RES DOI 10.1002/JBM.1217	2002	53	616
BRYANT SJ, 2004, J ORTHOP RES DOI 10.1016/J.ORTHRES.2004.02.001	2004	26	146
BURDICK JA, 2005, BIOMACROMOLECULES DOI 10.1021/BM049508A	2005	57	561
WILLIAMS CG, 2005, BIOMATERIALS DOI 10.1016/J.BIOMATERIALS.2004.04.024	2005	60	622
FEDOROVICH NE, 2009, BIOMATERIALS DOI 10.1016/J.BIOMATERIALS.2008.09.037	2009	43	287
JEON O, 2009, BIOMATERIALS DOI 10.1016/J.BIOMATERIALS.2009.01.034	2009	36	399
NICHOL JW, 2010, BIOMATERIALS DOI 10.1016/J.BIOMATERIALS.2010.03.064	2010	135	1396
CHEN YC, 2012, ADV FUNCT MATER DOI 10.1002/ADFM.201101662	2012	49	467
SHIN H, 2012, BIOMATERIALS DOI 10.1016/J.BIOMATERIALS.2011.12.050	2012	36	272
BILLIET T, 2014, BIOMATERIALS DOI 10.1016/J.BIOMATERIALS.2013.09.078	2014	24	610
ZHAO X, 2016, ADV HEALTHC MATER DOI 10.1002/ADHM.201500005	2016	25	447

In general, although it fluctuated in some years, the publications concerning photosensitive hydrogels for TE applications showed a steady upward trend as a whole. In terms of the number of publications, the United States was undoubtedly the leading country, with more articles published than the other 9 countries in the top 10 combined. Univ Colorado in the United States was the institution that published the most articles, and Anseth K from this institution was the most prolific author. An early article published by Anseth K group describing the applications of degradable photosensitive hydrogels based on poly(ethylene glycol) and poly(vinyl alcohol) in TE is also one of the landmark articles we extracted above(Anseth et al., 2002). Recently, the research group of Univ Colorado demonstrated the electrospun PEG fibrous hydrogels formed by thiol-ene photoclick chemistry and studied the effects of different process parameters on their properties (Sharma et al., 2021). Meanwhile, we could find that although China started this research not early, the growth rate of publications was relatively rapid, with Zhejiang Univ as its representative. In 2006, the research team from Zhejiang Univ developed a water-soluble chitosan hydrogel with photo crosslinking potential for the first time (Hong et al., 2006). Since then, the university has been committed to the study of photosensitive hydrogels for TE applications. Recently, the research group of Ouyang from Zhejiang Univ developed a biomimetic joint paint containing GelMA with hyaluronic acid which can be rapidly activated by light for the treatment of cartilage defect (Wei et al., 2021). However, as the two countries that published the most papers, the United States and China did not cooperate closely in this field. As we know, cooperation is an important means to promote the output of research results, whether between authors, institutions or countries. Therefore, China and the United States could collaborate more closely in photosensitive hydrogels for TE applications to promote the development of this research field. In addition, there was still a significant gap between developing countries and developed countries in this field, indicating that developing countries could invest more in this field and seek more opportunities for cooperation with developed countries.

In terms of the number of articles, the journal of Biomaterials had the most publications in this field by now, and it also published the most local cited references. For instance, the journal recently reported a Fluorescently LAbelled Sensitive Hydrogel (FLASH) generated by GelMA covalently bound to the FITC fluorophore, which can not only be fabricated into TE scaffolds by photo crosslinking, but also be used as a sensor to detect the degradation of the scaffolds during the process of new tissue formation (Onofrillo et al., 2021). The top 2 articles with the highest number of local citations and co-citations were all published in this journal. In addition, based on the analysis results of the journals publishing the articles, the journals publishing the cited documents, and the dual-map, we could know that the research topics in this field were mainly involved in (bio)materials science, chemistry, biology and medicine, and the application of photosensitive hydrogels for TE is an interdisciplinary field.

The keyword with the highest centrality was “extracellular matrix”, indicating the engineering ECM was the most important research content in this field. In addition, from the significant keywords and co-occurrence keywords in the above analysis we could know that, the main research scope in this field was to apply photosensitive hydrogels such as “alginate”-based, “hyaluronic acid”-based and “poly(ethylene) glycol”-based hydrogels to fabricate “tissue engineering scaffolds” (engineering ECMs) that meet the requirements, including the proper “mechanical property”, to study cell behaviors *in vitro*, such as the “differentiation” of “(mesenchymal) stem cell”. According to the results of keywords analysis, we could also identify that, “PEG hydrogel” and “poly(ethylene) glycol” were once the main research topics in this field. PEG-based photosensitive hydrogel is one of the most commonly used photopolymerizable synthetic materials and is widely used for TE and drug delivery applications (Yao et al., 2013; Qin et al., 2014; Jing et al., 2022; Liu et al., 2022). The analysis of keywords with citation bursts indicated that “gelatin” was a current research hotspot in this field, which is consistent with the results shown in trends of keyword occurrences map and the map of references clustering timeline. Gelatin is derived from type 1 collagen, which is the main component of mammalian ECMs (Van Hoorick et al., 2019). GelMA possessing photopolymerizable functionalities is formed by reacting the primary amines of hydroxylysine, lysine and ornithine with methacrylic anhydride. As a naturally-derived material, GelMA containing tripeptide arginine-glycine-aspartic acid (RGD) and matrix metalloproteinase (MMP) sequences has high bioactivity and biodegradability (Piao et al., 2021). As a hotspot in TE applications of photosensitive hydrogels in recent years, GelMA has attracted extensive study. For example, recently, Kumar et al. studied the effect of different GelMA synthesis parameters on the performance of the resulting bioink used in TE. Other research results on GelMA for TE applications are also numerous (Ovsianikov et al., 2011; Tytgat et al., 2018; Ying et al., 2018; Zhou et al., 2018; Kumar et al., 2021). Accordingly, the three publications recently appeared in the top 19 references with the strongest citation bursts are all reviews discussing the GelMA for TE applications.

It is worth noting that, “thiol-ene” was a research frontier in recent years, which is indicated in the map of references clustering timeline. As we all know, free-radical chain polymerization is a facile and easy-to-handle polymerization mechanism. However, chain growth has its inherent disadvantages, including 1) the multiple kinetic chains generating more heterogeneous networks; 2) the complex kinetic profile of polymerizations damaging the control of the reacted functionalities; 3) the oxygen inhibition resulting in longer processing times (Hoyle and Bowman 2010; Pereira and Bártolo, 2015; Tytgat et al., 2020). In contrast, thiol–ene click chemistry can form crosslinked polymer networks with a high degree of homogeneous structures at a very high reaction rate. Radical-mediated thiol-ene photo-click chemistry, which leads to step-growth polymerization for networks, is based on the extremely efficient reaction of thiols with non-homopolymerizable C=C double bonds, and it can surmount the disadvantages of chain-growth polymerization (Jing et al., 2022). Recently, Bilgik et al. demonstrated a clickable polyacrylamide hydrogel synthesized by photopolymerization using acrylamide and propargyl acrylate (Bilgic et al., 2014). Another article reported the synthesis and orthogonal crosslinking of poly(γ-glutamic acid)-norbornene photosensitive hydrogels and assesses the effect of the mechanical cues of hydrogels on the monocyte phenotype(Kim and Lin, 2021). It is noteworthy that the research groups of Vienna University of Technology and Ghent University has done a lot of research on thiol-ene hydrogels and processed them using two-photon polymerization technology to obtain homogeneous and high-resolution 3D structures for TE (Qin et al., 2013; Baudis et al., 2016; Van Hoorick et al., 2020). It is necessary to discuss the two-photon polymerization (TPP) technique here. Different from the traditional single photon polymerization, TPP uses a photoinitiator molecule in the photosensitive hydrogels to absorb two photons at the same time to initiate polymerization. The spatial resolution of the structures manufactured by TPP can reach below 100 nm. At present, it is the only technology capable of manufacturing complex 3D structures with precisely adjusted geometric shapes on a sub-cellular scale and is used to process engineering ECM (Fu et al., 2022).

The limitations of this study still exist. Firstly, analysis based on computer software would inevitably result in insufficient manual intervention. Secondly, although WoSCC is the most commonly used database for bibliometric analysis, it does not cover all articles in this field. Besides, articles after 1 October 2022 (the search end date) are excluded from this analysis. It should be pointed out here that there are other bibliometrics software based on different algorithms, such as VOSviewer, HistCite, *etc.* They can analyze scientific publications from different key directions. The software used in this article is powerful, but not perfect.

## Conclusion

This bibliometric and visualized study identifies the investigative information that are associated with photosensitive hydrogels for TE applications 1996-2022, using the bibliometric visualized tools CiteSpace and R-bibliometrix. Based off our analysis, we found that photosensitive hydrogels for TE applications is an interdisciplinary field widely studied, and its research achievements are expected to continue to grow steadily. The United States had absolute advantages in this field, and developing countries could seek opportunities for cooperation with it. The journal of Biomaterials published the most articles in this field, and Anseth K, Khademhosseini A and Bryant S had published lots of articles. Academic cooperation between authors, institutions and countries moves the development of this research field forward. Gelatin photosensitive hydrogels, especially GelMA-based hydrogels, have become a research hotspot in recent years, which is expected to last until the next years. In addition, as a research frontier, thiol–ene chemistry applied in photosensitive hydrogels for TE applications is expected to attract more researchers’ attention. The results of this study would provide scholars in relevant research fields with a profound understanding and insight into the applications of photosensitive hydrogels for TE.

## Data Availability

The original contributions presented in the study are included in the article/Supplementary material, further inquiries can be directed to the corresponding authors.
